# Genetic structure of SARS-CoV-2 reflects clonal superspreading and multiple independent introduction events, North-Rhine Westphalia, Germany, February and March 2020

**DOI:** 10.2807/1560-7917.ES.2020.25.22.2000746

**Published:** 2020-06-04

**Authors:** Andreas Walker, Torsten Houwaart, Tobias Wienemann, Malte Kohns Vasconcelos, Daniel Strelow, Tina Senff, Lisanna Hülse, Ortwin Adams, Marcel Andree, Sandra Hauka, Torsten Feldt, Björn-Erik Jensen, Verena Keitel, Detlef Kindgen-Milles, Jörg Timm, Klaus Pfeffer, Alexander T Dilthey

**Affiliations:** 1 Institute of Virology, University Hospital Düsseldorf, Heinrich Heine University Düsseldorf, Düsseldorf, Germany; 2 These authors contributed equally; 3 Institute of Medical Microbiology and Hospital Hygiene, Heinrich Heine University Düsseldorf, Düsseldorf, Germany; 4 Department of Gastroenterology, Hepatology and Infectious Diseases, University Hospital Düsseldorf, Heinrich Heine University Düsseldorf, Düsseldorf, Germany; 5 Department of Anaesthesiology, University Hospital Düsseldorf, Heinrich Heine University Düsseldorf, Düsseldorf, Germany

**Keywords:** COVID-19, genomic epidemiology, Heinsberg, Düsseldorf, artic, nanopore, superspreading, SARS-CoV-2

## Abstract

We whole-genome sequenced 55 SARS-CoV-2 isolates from Germany to investigate SARS-CoV-2 outbreaks in 2020 in the Heinsberg district and Düsseldorf. While the genetic structure of the Heinsberg outbreak indicates a clonal origin, reflecting superspreading dynamics from mid-February during the carnival season, distinct viral strains were circulating in Düsseldorf in March, reflecting the city’s international links. Limited detection of Heinsberg strains in the Düsseldorf area despite geographical proximity may reflect efficient containment and contact-tracing efforts.

We report on the genetic structure of severe acute respiratory syndrome coronavirus 2 (SARS-CoV-2) in North-Rhine Westphalia, Germany’s most populous state (18 million inhabitants). Our analysis includes the ‘Heinsberg outbreak’ [1], which started in the second half of February 2020 – comprising a superspreading event at a carnival session in Gangelt, a small municipality of ca 12,000 inhabitants on the border between Germany and the Netherlands – and subsequent outbreak dynamics in March, in the state capital Düsseldorf, located 70 km from Gangelt and an international economic and air travel hub of ca 600,000 inhabitants.

## Severe acute respiratory syndrome coronavirus 2 genome sequencing

The institute of virology at Düsseldorf University Hospital was one of the first laboratories to offer SARS-CoV-2 diagnostics in North-Rhine Westphalia. A total of 55 SARS-CoV-2 isolate samples were acquired from diagnostic swabs sent to this institute in February and March 2020. Of these, 10 were directly linked to the Heinsberg outbreak (obtained from medical practices in the Heinsberg district or from patients treated at Düsseldorf University Hospital who were Heinsberg district residents) and 45 originated from the city of Düsseldorf and surrounding districts.

RNA extraction and reverse transcription were carried out as previously described [2]. DNA amplification and sequencing on the Oxford Nanopore platform were carried out according to the Artic protocol [3,4] (Supplementary Text), yielding between 31 and 582 Mb of raw sequencing data per sample (Supplementary Table S1). Bioinformatic analysis was based on the Artic pipelines and additional manual curation was carried out (Supplementary Text), yielding completely resolved genomes with 2–13 polymorphic positions (Supplementary Table S2) relative to the SARS-CoV-2 reference genome [5].

Of note, we observed evidence for ambiguities at polymorphic positions in 11 of 55 samples (Supplementary Table S2); for one such sample (NRW-39; 13 positions called as multi-allelic), PCR was repeated and a separate sequencing run was carried out, confirming the detected ambiguities (Supplementary Text). Further work is necessary to investigate whether ambiguities represent within-patient viral quasispecies.

In a proof-of-concept experiment, we also successfully sequenced reverse-transcribed viral cDNA from patient material without an intermediate PCR-based amplification step (Supplementary Text), potentially enabling simplified sample preparation and increased read lengths for some samples in the future.

## Ethical statement

Our study was Institutional Review Board (IRB)-approved by the ethics committee of the Heinrich Heine University Düsseldorf (#2020–839).

## The Heinsberg outbreak

The first cases of SARS-CoV-2 infection in Germany were detected in late January 2020 and could be linked to recent travel to Northern Italy and China [1]. On 24 and 25 February 2020, however, two members of the same household from the Heinsberg district with no known travel history to SARS-CoV-2 risk areas were diagnosed with SARS-CoV-2; by 28 February 2020, the number of confirmed infections in the Heinsberg district had grown to 37; by 22 April 2020, to > 1,700 [6]. Contact tracing later showed that many of the early SARS-CoV-2 cases could be linked to a carnival session attended by the two index cases. The carnival event was held on 15 February 2020 in the municipality of Gangelt, which is part of the Heinsberg district [1]. Epidemiological investigation revealed that the index cases had travelled to the Netherlands, not considered a risk area at the time, 7 days prior to attending the Gangelt carnival session. The ‘Heinsberg outbreak’ represented one of the first large-scale SARS-CoV-2 outbreaks in Germany, seeded by community transmission and amplified by superspreading-type dynamics.

Genomic analysis of 10 SARS-CoV-2 isolates from the Heinsberg outbreak, sampled between 25 and 28 February and including those from the index cases, demonstrated the clonal origin of the outbreak (Figure); all Heinsberg samples shared the same two mutations compared to the SARS-CoV-2 reference genome (Supplementary Table S2). Viral diversity in the Heinsberg samples varied between two and six polymorphic positions relative to the SARS-CoV-2 reference genome, and five distinct viral variants (i.e. haplotypes) could be identified (Supplementary Table S2).

**Figure:**
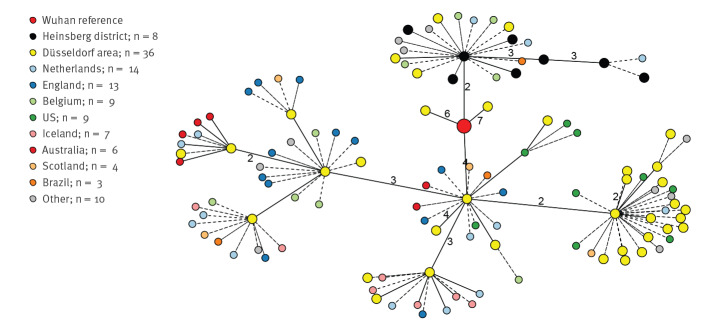
Minimum spanning tree of severe acute respiratory syndrome coronavirus 2 sequences, showing 44 unambiguously^a^ resolved genomes from the Heinsberg district (n = 8) and the Düsseldorf area (n = 36), Germany, February–March 2020

GISAID: Global Initiative on Sharing All Influenza Data; SARS-CoV-2: severe acute respiratory syndrome coronavirus 2; US: United States.

^a^ Eleven isolate genomes from 55 in our study with ambiguities are not considered ‘unambiguously resolved’ for the purposes of this analysis and are thus omitted.

The original Wuhan SARS-CoV-2 reference genome; and a sample of closely related publicly available SARS-CoV-2 genomes from GISAID (distance to any of the Heinsberg/Düsseldorf genomes of 0 or 1; see Supplementary Text for details) are included in the analysis.

Dashed and solid edges without adjacent numbers indicate distances of 0 and 1, respectively; all other distances are shown explicitly.

An analysis (Supplementary Text) of other publicly available SARS-CoV-2 sequences did not reveal an obvious origin of the Heinsberg outbreak (Supplementary Table S3); the Heinsberg isolates are not related to early sequences from other German outbreak areas (Bavaria, Baden-Wuerttemberg), and, despite intense Dutch viral sampling (585 available viral genomes from the Netherlands at the time of analysis), our analysis identified only two closely related isolates from the Netherlands (one collected on 21 March, the other with undefined collection date). The role of the index cases’ short vacation in the Netherlands 7 days before the Gangelt carnival session [7], while suggestive in terms of reported SARS-CoV-2 incubation periods thus remains ambiguous [8]. Moreover, large numbers of closely related isolates are circulating in many countries, for example England, Wales, and Iceland (Supplementary Table S3). The small number of polymorphisms shared by all samples in the Heinsberg outbreak (n = 2), compared with a maximum number of six per-isolate polymorphic positions in the same samples, likely acquired over a period of a few weeks, is compatible with a relatively recent introduction from China.

## Düsseldorf outbreak dynamics

The first SARS-CoV-2 cases in Düsseldorf, 70 km from Gangelt, were diagnosed in early March 2020 [9]; as at 21 April 2020, the outbreak had grown to more than 900 confirmed cases [10]. The set of 55 whole-genome-sequenced isolates included 45 samples from Düsseldorf and nearby districts, collected between 3 and 23 March. A minimum spanning tree analysis of 44 unambiguously resolved viral sequences (Figure) showed that there were at least five clusters of viral stains circulating in the Düsseldorf area; the number of polymorphic positions relative to the SARS-CoV-2 reference genome in the Düsseldorf samples varied between 2 and 13 (Supplementary Table S2). Closely related strains (distance 0 or 1) were found in Australia, the United Kingdom, the United States and many other countries (Supplementary Table S3), strongly suggesting multiple independent introduction events. Of note, four ‘Düsseldorf area’ isolates clustered with the Heinsberg outbreak (Figure 1); of these, two were collected from residents of a district next to Heinsberg, who had been treated at the Düsseldorf University Hospital, and two remained of unclear origin (patient data not available). Thus, there was no evidence for widespread community circulation of Heinsberg-derived SARS-CoV-2 strains in the Düsseldorf area.

## Illumina validation

To verify the accuracy of Nanopore-based viral assembly, additional Illumina sequencing was carried out for the first 11 samples, according to date of collection, of our cohort (Supplementary Table S1; Supplementary Text); data analysis was carried out with iVar [11]. For 41 of 45 polymorphic positions identified by either Nanopore or Illumina across the 11 samples, the called alleles agreed; manual inspection of the discordant positions revealed low coverage for two discordant positions and one missed multi-allelic call for each sequencing technology (Supplementary Table S4).

## Discussion

Since its emergence in the Chinese city of Wuhan in late 2019, SARS-CoV-2 has infected more than 6 million individuals and led to more than 370,000 deaths worldwide as at 03 June 2020 [12]. As SARS-CoV-2 case numbers and the social and economic consequences of social distancing and lock-down measures continue to rise, many countries are facing difficult trade-offs. Improved methods to characterise the dynamics of viral transmission are urgently needed.

More than 10,000 globally sourced SARS-CoV-2 genomes are publicly available, and powerful data sharing and analysis platforms like the Global Initiative on Sharing All Influenza Data (GISAID) EpiCoV database [13] and Nextstrain [14] enable the collaborative analysis of viral population structure on a global level. Additional insights into transmission dynamics can be gained from focused investigations of individual outbreaks and by integrating genomic data with classical epidemiology.

Here we have investigated the genetic structure of two SARS-CoV-2 outbreaks, which occurred at two nearby locations in North-Rhine Westphalia using Nanopore sequencing, which has additional applications in many fields such as human genetics [15] and microbial metagenomics [16]. We have demonstrated the clonal origin of the Heinsberg outbreak. This is consistent with available epidemiological data pointing to a carnival session in Gangelt as the epicentre of the outbreak [1]. The lack of association between the Heinsberg samples and other early German outbreak isolates is suggestive of a separate introduction event, possibly via the Netherlands, China, or a third country. By contrast, SARS-CoV-2 isolates circulating in Düsseldorf were highly polyclonal and could be grouped into at least five clusters of viral haplotypes.

Despite the geographical proximity between Heinsberg and Düsseldorf, only four of 36 unambiguously resolved samples from the Düsseldorf area clustered with the Heinsberg outbreak, and two of these were derived from residents of a district neighbouring Heinsberg. Limited detection of Heinsberg strains in the Düsseldorf area may reflect the effectiveness of the contact-tracing efforts conducted by the German public health authorities; of note, ‘lockdown’-type restrictions with limits on public gatherings in Germany were only imposed on 23 March 2020 [17], i.e. on the day on which the last sample of our study was collected.

More extensive sampling of SARS-CoV-2 isolates from North-Rhine Westphalia will be required to investigate the effect of various containment measures on transmission chains at a genomic level. Consistent with reports from Iceland [18], New York [19], and data on Nextstrain, our study has demonstrated the simultaneous circulation of distinct viral variants (i.e. haplotypes) in a metropolitan region. In the Heinsberg outbreak, we could identify five distinct variants. As SARS-CoV-2 genomes continue to diverge as part of ongoing viral evolution, the application of genomic epidemiology [20,21] for the identification and targeted interruption of viral transmission chains will become increasingly feasible.

## Data availability

All generated viral genome assemblies have been submitted to GISAID; all generated assemblies and the raw sequencing data are also available on NCBI (BioProject PRJNA627229).

## Supplementary Data

1168000 Supplementary Text

## Supplementary Data

26000 Supplementary Table S1

## Supplementary Data

40000 Supplementary Table S2

## Supplementary Data

59000 Supplementary Table S3

## Supplementary Data

23000 Supplementary Table S4

## Supplementary Data

781000 Supplementary Table S5
