# Supplementary material for: Genetic structure of SARS-CoV-2 reflects clonal superspreading and multiple independent introduction events, North-Rhine Westphalia, Germany, February and March 2020
Source: Euro Surveill. 2020 Jun 4;25(22):2000746. doi: 10.2807/1560-7917.ES.2020.25.22.2000746 (PMC7336109; doi:10.2807/1560-7917.ES.2020.25.22.2000746)
Supplement: Supplementary Text [file 2000746_DILTHEY_Supplementary_Text.pdf]

This supplementary material is hosted by Eurosurveillance as supporting information alongside the article '*Genetic structure of SARS-CoV-2 reflects clonal superspreading and multiple independent introduction events, North-Rhine Westphalia, Germany, February and March 2020*', on behalf of the authors, who remain responsible for the accuracy and appropriateness of the content. The same standards for ethics, copyright, attributions and permissions as for the article apply. Supplements are not edited by Eurosurveillance and the journal is not responsible for the maintenance of any links or email addresses provided therein.

Supplementary Text

RNA extraction and Nanopore sequencing

Respiratory samples from nasopharyngeal swabs were used for total nucleic acid extraction using the EZ1 Virus Mini Kit v2.0 on an EZ1 Advanced XL (Qiagen, Germany) according to manufacturers' instructions. SARS-CoV-2 was detected as described[1] with a plasmid-standard for quantification. Viral RNA was reverse-transcribed to single-strand cDNA using random hexamers and SuperScript[2]. Viral cDNA was PCR-amplified using the Artic network SARS-CoV-2 protocol with V1 primers[3,4]. Prior to library preparation, for each sample, PCR pools 1 and 2 were combined (500ng DNA per pool). Sequencing was carried out on the Oxford Nanopore Flongle and MinION devices (Supplementary Table S1), utilizing FLO-FLG001 and FLO-MIN106 flow cells and the SQL-LSK109 ligation sequencing kit. On the MinION device, barcoding was carried out with the EXP-NBD104 and EXP-NBD114 barcoding kits (Supplementary Table S1). Flongle runs were carried out without multiplexing.

Bioinformatic analysis

Nanopore sequencing reads were basecalled with Guppy 3.4.5+fb1fbfb and analysed with the Nanopolish-[5] and Medaka-based workflows provided by Artic (https://github.com/artic-network/artic-ncov2019.git, commit ddfb2dc87a4f442f821787ef90a92625f6bd6a09).

The pipeline comprises the following steps:

- Length filtering of the generated reads to identify and remove reads that emanate from chimeric PCR fragments.

```
artic gather --min-length 300 --max-length 700 --directory
out_dir
```

- Demultiplexing with porechop v0.3.2[6] . The software automatically determines the utilized adapter sets und bins the reads according to their barcode with a lower limit of at least 80% identity.

```
porechop --verbosity 2 --untrimmed -i input_file -b output_dir
--native_barcodes --discard_middle --require_two_barcodes --
barcode_threshold 80 --check_reads 10000 --barcode_diff 5 >
input_file.demultiplexreport.txt
```

- Mapping, coverage normalisation (target 200x) and adapter trimming with BWA-MEM[7]

```
bwa mem -x ont2d reference.fasta input_reads.fastq | samtools
view -bS - | samtools sort -o input_reads.sorted.bam -
```

and the Artic script align_trim:

```
align_trim –normalise 200 primers.bed  <
input_reads.sorted.bam 2> input_reads.alignreport.er |
samtools view -bS - | samtools sort - -o
out.primertrimmed.sorted.bam
```

- Variant calling and consensus generation with Medaka, if using the Medaka workflow:

```
medaka consensus out.primertrimmed.sorted.bam out.hdf
medaka snp  reference.fasta out.hdf
out.primertrimmed.medaka.vcf

margin_cons_medaka --depth 20 --quality 10 reference.fasta
out.primertrimmed.medaka.vcf out.primertrimmed.sorted.bam >
consensus.fasta 2> medaka.report.txt
```

- Variant calling and consensus generation with Nanopolish, if using the Nanopolish workflow:

```
nanopolish variants --min-flanking-sequence 10 --fix-
homopolymers -x 1000000 --progress --reads nanopolish.index -o
out.primertrimmed.vcf -b out.primertrimmed.sorted.bam -g
reference.fasta -w nanopolish_header --snps --ploidy 1 -m 0.1

margin_cons reference.fasta out.vcf
out.primertrimmed.sorted.bam a > nanoplish.consensus.fasta
```

- By default, genome positions with less than 20x coverage are masked with 'N' characters by the Medaka- and Nanopolish-based consensus generation workflows.

The generated VCF files and consensus FASTA sequences were manually curated by a) carrying out a comparison between the Nanopolish- and Medaka-based VCF files and b) visual inspection with IGV[8], checking for i) false-positive calls; ii) polymorphic positions with more than one plausible allele; iii) missed calls (false negatives).

Across the cohort, we identified the following recurrent error modes: i) Medaka over-called polymorphisms in low-coverage regions; these are automatically masked out by the ARTIC pipeline; ii) in rare instances, adapter sequences were not removed completely by the ARTIC pipeline preprocessing steps, leading to clustered false-positive allele calls in Medaka, but not in Nanopolish; iii) in rare instances, Nanopolish detected likely false-positive polymorphisms not called by Medaka and exhibiting strong strand bias; iv) potentially multi-allelic positions were, though sometimes identified by Medaka as 'heterozygous', often missed by both callers.

For each sample, a final consensus FASTA file was produced, in which i) regions with false-positive calls were masked out with 'N' characters; ii) ambiguous or potentially multi-allelic positions were represented with the corresponding IUPAC ambiguity codes.

Direct cDNA viral sequencing

For a proof-of-concept experiment, we selected a sample (NRW-39) with high viral load and carried out reverse transcription (RT) as described above, using a) random hexamer primers and b) a subset of the Artic primers. The resulting single-stranded cDNA pools were converted into double-stranded DNA with the NEBNext® Ultra™ II Non-Directional RNA Second Strand Synthesis Module (NEB) and sequenced on separate Flongle flow cells as described above. The generated sequencing reads were basecalled as described above and aligned with minimap2[9] (version 2.17-r974-dirty, flags `-a -x map-ont`) against a combined FASTA file containing the GRCh38 version of the human reference genome[10] and the SARS-CoV-2 reference genome (MN908947.3)[11]. All generated sequencing data were submitted to SRA (Sequencing Read Archive; Supplementary Table S1).

Manual inspection with IGV[8] showed that the alleles of the polymorphic positions called based on the standard Artic protocol (bottom panel in the following figure) were recovered from the direct cDNA sequencing data based on RT primed with Artic primers (top panel in the following figure); the number of positions with low-frequency non-consensus alleles, however, was increased when compared to the standard Artic run (we note, however, that this might also be due to slightly increased error rates on the Flongle platform compared to the MinION).

By contrast, the data generated based on random RT priming exhibited significantly increased noise levels (middle panel in the following figure).

We conclude that further work, including on samples with lower viral load, is required to assess the potential of direct cDNA sequencing from SARS-CoV-2 patient material.

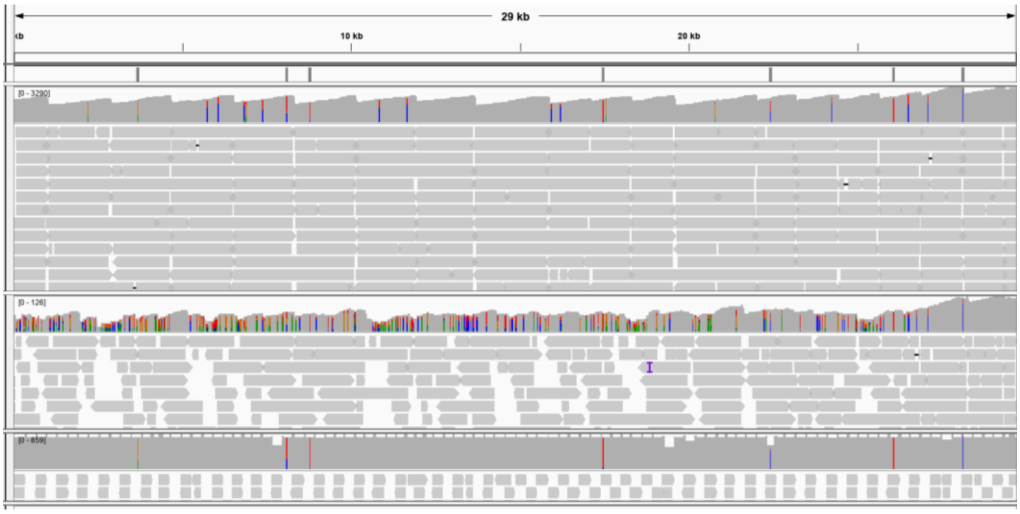

IGV-based comparison of direct cDNA and PCR-based sequencing data and variant calls on sample NRW-39. Panels, from top to bottom: Medaka VCF, based on Artic PCR; coverage and reads, RT primed with Artic primers; coverage and reads, RT primed with random hexamers; coverage and reads, Artic PCR. Note that the sequencing data in the upper two panels were generated on the Oxford Nanopore Flongle platform, which typically exhibits slightly higher error rates than the MinION platform, which was used for the generation of the sequencing data visualized in the bottom panel.

Illumina sequencing and bioinformatics analysis

To verify the accuracy of the Nanopore-based viral assemblies, viral genome sequencing was repeated for the first 12 isolates of our cohort (Supplementary Table S1). DNA amplicon samples, generated with the Artic protocol as described above, were quantified by fluorometric assay (Qubit DNA HS Assay, Thermo Fisher Scientific) and quality was assessed by capillary electrophoresis using the

Fragment Analyzer and the "High Sensitivity NGS Fragment Kit (1-6000bp) Assay" (Agilent Technologies, Inc. Santa Clara, USA). All samples showed high quality amplicons with 400bp fragment length. Library preparation was performed using the "TruSeq Nano DNA Library Prep Kit - Part # 15041110 Rev. D protocol" (Illumina Inc. San Diego, USA) by skipping the fragmentation step and starting with the repair ends steps. Briefly, 200 ng of each sample was used for end repair, overhang conversion, adenylation, adapter ligation and library amplification. Bead purified libraries were normalized and finally sequenced on the MiSeq system (Illumina Inc. San Diego, USA) with a read setup of 2x251bp. The bcl2fastq tool was used to convert the bcl files to fastq files as well for adapter trimming and demultiplexing. Bioinformatic analysis was based on iVar[12] (v 1.0.1). The generated assemblies (consensus FASTA sequences) were compared to the Nanopore-based assemblies and positions with discrepant alleles were assesses with IGV[8] (Supplementary Table S3).

Genomic neighbourhood analysis and minimum spanning tree computation

A genomic neighbourhood analysis, as defined below, was carried out for all isolate sequences present in GISAID[13] as of 18 April 2020, including the Düsseldorf/Heinsberg sequences generated as part of this study.

1. All sequences in GISAID were aligned against the SARS-CoV-2 reference genome (GenBank: MN908947.3). Isolate sequences with ambiguous characters, excluding 'N', and sequences longer than 29,903bp, were excluded from further analysis. Pairwise alignments were computed with edlib[14] in ends-free alignment mode. If the first or last 10 alignment columns contained any gaps, the isolate was excluded from further analysis. If necessary, the pairwise alignment was padded with 'N' characters to cover the complete reference sequence.
2. For each remaining isolate sequence, the pairwise alignment was converted into a list of polymorphic positions relative to the reference genome by determining, for each nucleotide of the reference sequence, the allele present in the isolate sequence (in this representations, consecutive single-nucleotide polymorphisms or deletions relative to the reference are represented as separate reference-divergent alleles, whereas consecutive insertions are represented as one reference-divergent allele). 'N' alleles were discarded.
3. A pairwise distance matrix was computed for all remaining isolate sequences, by determining, for each pair of isolate sequences, the number of non-shared polymorphic alleles
4. For each Düsseldorf/Heinsberg isolate sequence part of the distance matrix, up to 10 "neighbouring" isolate sequences were identified from the set of other isolates sequences, employing the following steps: a) the identifiers of all samples present in the computed distance matrix were stored as a vector V, excluding any Düsseldorf/Heinsberg samples; b) the order of V was randomized; c) for each Düsseldorf/Heinsberg sequence identifier x, a vector V' was generated by stably sorting the elements of V according to their distance to x in increasing order; d) V' was filtered to exclude any elements with distance to x of more than 1; e) the first 10 elements of V' were chosen as the neighbours of x (if there were fewer than 10 elements in V', all elements of V' were chosen as the neighbours of x). The output of step d) is shown in Supplementary Table S4, i.e. the table shows, for all Düsseldorf/Heinsberg sequences, the IDs and country-level statistics of all possible neighbour sequences.
5. A minimum spanning tree analysis was carried out for all Düsseldorf/Heinsberg sequences part of the distance matrix, and their selected genomic neighbours, using the `mst` function of the R[15] package pegas[16] and visualized with functions from sna[17].

References

1. Corman, V.M. *et al.* Detection of 2019 novel coronavirus (2019-nCoV) by real-time RT-PCR. *Euro Surveill* **25**(2020).
2. Walker, A., Ennker, K.S., Kaiser, R., Lubke, N. & Timm, J. A pan-genotypic Hepatitis C Virus NS5A amplification method for reliable genotyping and resistance testing. *J Clin Virol* **113**, 8-13 (2019).
3. Quick, J. ARTIC amplicon sequencing protocol for MinION for nCoV-2019. https://dx.doi.org/10.17504/protocols.io.bbmuik6w *(2020)*
4. Quick, J. *et al.* Multiplex PCR method for MinION and Illumina sequencing of Zika and other virus genomes directly from clinical samples. *Nat Protoc* **12**, 1261-1276 (2017).
5. Loman, N.J., Quick, J. & Simpson, J.T. A complete bacterial genome assembled de novo using only nanopore sequencing data. *Nat Methods* **12**, 733-5 (2015).
6. Wick, R.R., Judd, L.M., Gorrie, C.L. & Holt, K.E. Completing bacterial genome assemblies with multiplex MinION sequencing. *Microb Genom* **3**, e000132 (2017).
7. Li, H. Aligning sequence reads, clone sequences and assembly contigs with BWA-MEM. in *ArXiv e-prints* Vol. 1303 (2013).
8. Robinson, J.T. *et al.* Integrative genomics viewer. *Nat Biotechnol* **29**, 24-6 (2011).
9. Li, H. Minimap2: pairwise alignment for nucleotide sequences. *Bioinformatics* **34**, 3094-3100 (2018).
10. Schneider, V.A. *et al.* Evaluation of GRCh38 and de novo haploid genome assemblies demonstrates the enduring quality of the reference assembly. *Genome Res* **27**, 849-864 (2017).
11. Wu, F. *et al.* A new coronavirus associated with human respiratory disease in China. *Nature* **579**, 265-269 (2020).
12. Grubaugh, N.D. *et al.* An amplicon-based sequencing framework for accurately measuring intrahost virus diversity using PrimalSeq and iVar. *Genome Biol* **20**, 8 (2019).
13. Shu, Y. & McCauley, J. GISAID: Global initiative on sharing all influenza data - from vision to reality. *Euro Surveill* **22**(2017).
14. Sosic, M. & Sikic, M. Edlib: a C/C ++ library for fast, exact sequence alignment using edit distance. *Bioinformatics* **33**, 1394-1395 (2017).
15. R Core Team. R: A Language and Environment for Statistical Computing. (2019).
16. Paradis, E. pegas: an R package for population genetics with an integrated-modular approach. *Bioinformatics* **26**, 419-20 (2010).
17. Butts, C.T. Social Network Analysis with sna. *Journal of Statistical Software* **24**, 51 (2008).
